# The effect of varicocele repair for sperm DNA fragmentation: A protocol for systematic review and meta-analysis

**DOI:** 10.1097/MD.0000000000021960

**Published:** 2020-09-18

**Authors:** Hangyu Yao, Fuhao Li, Xianliang Qiu, Yuanjie Xu, Pengfei Xue, Degui Chang

**Affiliations:** Hospital of Chengdu University of Traditional Chinese Medicine, Chengdu, Sichuan Province, China.

**Keywords:** DNA fragmentation index, sperm DNA damage, sperm DNA fragmentation, varicocele, varicocele repair

## Abstract

**Introduction:**

Sperm DNA integrity has been considered as one of the important determinants of normal fertilization and embryonic development in natural and assisted pregnancy. It is difficult for men with high levels of sperm DNA fragmentation (SDF) in semen to conceive their partners naturally and assist in conception. The studies have found that the level of SDF in the semen of patients with varicocele (VC) was on the high side. In recent years, the effect of VC surgery on DNA fragmentation index has attracted the attention of researchers. In this study, we will evaluate the effectiveness of VC repair as a way to alleviate SDF and improve male fertility.

**Methods and analysis:**

Electronic databases including English databases (PubMed, MEDLINE, EMBASE, Web of Science, Cochrane Library) and Chinese databases (China National Knowledge Infrastructure, China Biology Medicine Database, Wanfang Database, VIP Database) will be searched from their inception to December 2020 to recognize related studies. All the randomized controlled trials of microsurgical varicocelectomy for the management of VC patients will be included. The potential outcome will include improvement in SDF, oxidative stress markers (reactive oxygen species, nitric oxide, and lipid peroxidation products), sperm chromatin compaction, other advanced sperm function characteristics, follow-up of fertility results. We will conduct this study strictly according to the Cochrane Handbook for Systematic Reviews of Interventions.

**Results:**

The study is a protocol for systematic review and meta-analysis without results, and data analysis will be carried out after the protocol. We will share our findings on April 5th of 2021.

**Conclusion:**

This systematic review will provide more evidence to assess whether varicocelectomy is an effective intervention for patients with SDF. The results will be published in a public issue journal and offer the urologists help to make clinical decisions.

**Ethics and dissemination:**

Formal ethical approval is not required in this protocol. We will collect and analyze data based on published research. Since this research does not involve patients, personal privacy will not be affected. The results of this review will be distributed to peer-reviewed journals or submitted to relevant conferences.

**Protocol registration number:**

INPLASY202070119

## 1 Introduction

Sperm DNA is found to be very important for the development of healthy embryos in existing studies.^[1,2]^ And it is difficult for men with high levels of Sperm DNA fragmentation (SDF) in semen to conceive their partners naturally and assist in conception. Among couples who became pregnant without assistance, male couples with high SDF were more likely to be pregnant for longer.^[3,4]^

At present, more and more attention has been paid to the relationship between varicocele (VC) and SDF. VC consists of abnormal dilatation of discoid plexus veins. It is commonly seen in the general male population, affecting 15% of individuals of childbearing age, 35% of patients with primary infertility, and up to 80% of secondary infertile men.^[5–7]^ More and more attention has been paid to the relationship between VC and SDF. VC consists of abnormal dilatation of discoid plexus veins. It is commonly seen in the general male population, affecting 15% of individuals of childbearing age, 35% of patients with primary infertility, and up to 80% of secondary infertile men.^[8]^ Studies have shown that reactive oxygen species and apoptosis markers are increased in the semen of infertile men with VC. The imbalance between the production of reactive oxygen species and antioxidant protection leads to oxidative stress, which can damage lipids, proteins, and nucleic acids in living sperm.^[9]^ At the same time, oxidative stress can destroy the chromatin structure of spermatozoa by inducing DNA chain breakage, which in turn affects the reproductive function of men.^[10–12]^ DNA fragmentation index (DFI) is a highly stable index, some patients with VC sperm motility density and morphological analysis can be in the normal range, but sperm DFI is significantly higher than normal.

Surgical treatment of infertility with VC has a history of more than a century.^[13]^ In fact, the use of surgical treatment of VC is associated with significant improvements in various semen parameters of male infertility.^[14,15]^ Recently, VC repair has been used to treat male infertility and high SDF caused by spontaneous VC.^[16]^ There are many clinical trials related to VC surgery and DFI. In this study, we will evaluate the effectiveness of VC repair as a way to alleviate SDF and improve male fertility.

## 2 Objectives

With this systematic review and if possible meta-analysis we urge to further evaluate the effectiveness of VC repair as a way to alleviate SDF and improve male fertility. The results will offer clinical decisions for urologists and andrologists. So far, the meta-analysis about the effect of VC repair for SDF was published in 2012, which only included 6 studies, suggested that varicocelectomy may be a possible treatment for SDF; however, more studies with appropriate controls are needed to confirm this finding. Further investigation is warranted given that an increasing number of studies about the effects of VC repair for SDF have been carried out in recent years. Therefore, we will conduct an up-to-date systematic review and meta-analysis for existing randomized controlled trials (RCTs) with the aim of further assessing the effectiveness of VC repair as a way to alleviate SDF and improve male fertility.

## 3 Methods

The protocol was registered on the International Platform of Registered Systematic Review and Meta-analysis Protocols (registration number:INPLASY202070119) which could be available on https://inplasy.com. The content refers to the statement of the Preferred Reporting Items for Systematic Review and Meta-Analysis Protocols (PRISMA-P) checklist.^[17,18]^

### 3.1 Eligibility criteria

The inclusion and exclusion criteria are as follows.

#### 3.1.1 Types of studies

All the RCTs of patients with VC were treated by surgery and SDF was detected before and after the operation will be included without publication status restriction or writing language. letters to editors, review articles, case reports, conference abstracts, cross-sectional studies, and all observational studies will be excluded. Because this is surgical treatment, so it is difficult to achieve random, controlled. As long as the criteria of PRISMA are met, relevant clinical trials can be systematically reviewed and meta-analysis can be conducted if necessary. Therefore, some other suitable research types can be included.

#### 3.1.2 Participants

Inclusion criteria:

- Patients have been diagnosed with VC by physical examination and color Doppler ultrasonography of the male reproductive system.
- Patients who were tested before the operation and showed high SDF

Exclusion criteria:

- Patients with a history of scrotal and spermatic cord injuries and congenital genitourinary abnormalities.
- Patients who have been operated on for VC.
- Patients with a history of tumors and diabetes in the past year.
- Patients with any other disease that may cause VC (such as external kidney tumor, hydronephrosis, etc)

#### 3.1.3 Types of interventions and controls

*Experimental interventions:*

The patients in the treatment group received varicocelectomy (no restriction on the methods of operation and course of treatment).

*Control interventions:*

The control group could gain a placebo, no treatment, exercise, or guideline-recommended conventional treatment.

#### 3.1.4 Types of outcome measures

*Primary outcome:*

Improvement in sperm DFI. [The sperm chromatin structure assay [SCSA], terminal deoxynucleotidyl transferase dUTP nick end labeling [TUNEL], sperm chromatin dispersion test [SCD], and single gel electrophoresis [Comet] are the most commonly used methods to measure SDF^[19,20]^)

Secondary outcomes:

(1) oxidative stress markers (Reactive oxygen species, nitric oxide, and lipid peroxidation products)
(2) sperm chromatin compaction
(3) other advanced sperm function characteristics
(4) Follow-up of fertility results.

### 3.2 Search strategy

#### 3.2.1 Data sources

Electronic databases including English databases (PubMed, MEDLINE, EMBASE, Web of Science, Cochrane Library) and Chinese databases (China National Knowledge Infrastructure, China Biology Medicine Database, Wanfang Database, VIP Database) will be searched from their inception to December 2020 to recognize related studies. The search strategy that will be run in the PubMed and tailored to the other database when necessary is presented in Table 1. Besides, the reference lists of review articles will be searched for any possible titles matching the inclusion criteria.

**Table 1:**
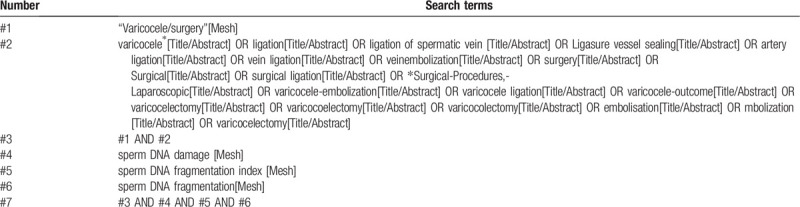
This table presents the initial draft of the search strategy with PubMed as an example.

#### 3.2.2 Other sources of search

The researchers will also scan the database of Chengdu University of Traditional Chinese Medicine Library and consult the experts in urology. Dissertations of degrees will be included. The WHO International Clinical Trials Registry Platform and Google Scholar will be scrutinized for potential results. In addition, the ClinicalTrials.govregistry will be explored to find any unpublished trials.

### 3.3 Data extraction, quality, and validation

#### 3.3.1 Study inclusion

**Figure 1:**
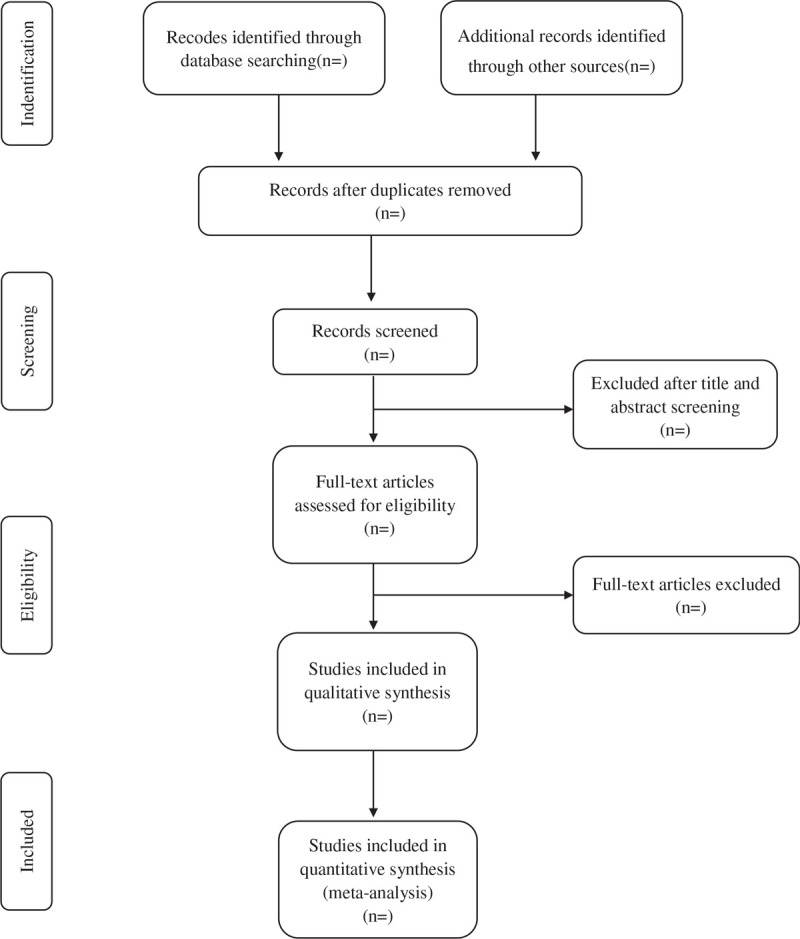
Study selection flow chart.

According to predefined eligibility criteria, researchers will import the literature retrieved to the Endnote X8 and eliminate the duplicate data. Studies will be removed if they do not meet the inclusion criteria. If the studies appear to meet the inclusion criteria or there is any uncertainty based on the information provided in the title and abstract, full texts will be obtained for further assessment. When necessary, we will contact the author for more details of the study to solve questions about eligibility. Two researchers will independently conduct the literature search and literature screening. Disagreements will be resolved by discussion or taking the expert (DGC) for arbitration. The number and reasons for excluding trials will be recorded in detail. A flow diagram of the study selection is shown in Figure 1.

#### 3.3.2 Data extraction and management

Upon completion of the retrieval, the 2 reviewers will independently read and extract the data from the study. Data will include the following information: title, abstract, first author and corresponding author, the country, the publication time, publications, participants, demographic characteristics (age, baby and family situation, regional, ethnic, and national), the number of participants, diagnostic criteria, types, intervention, intervention characteristics (incision length, unilateral or bilateral), observation index (sperm DFI, oxidative stress markers, sperm chromatin compaction and other advanced sperm function characteristics), the results of the study, the incidence of adverse events and type. We will use a standardized data extraction table to extract the above data. Any disagreement between the 2 reviewers will be decided by consensus or with the participation of a third reviewer. Finally, we will contact the author via email to request any missing data or clarification. If we cannot obtain the missing data, we will report it in the risk assessment of bias and consider its impact on the analysis of the data.

### 3.4 Risk of bias assessment

The risk of bias will be independently assessed by 2 reviewers and any differences will be resolved through consultation or the participation of a third reviewer. The RCTs will be evaluated using the Cochrane “risk of bias assessment” tool. The tool assesses the risk of bias mainly in the following 7 aspects: random sequence generation, allocation concealment, the blinding method for patients, researchers and outcomes assessors, incomplete result data, and selective reports. As recommended by the Cochrane manual, the risk of bias in each of these areas will be assessed as low or high depending on whether the criteria were met or not met, and the lack of information will be recorded as unclear. In most cases, disagreements will be settled by discussion between the 2 reviewers. If disagreement remained after discussion, a third reviewer will be consulted before taking the final decision on the disagreements.

### 3.5 Quantitative data synthesis and statistical methods

#### 3.5.1 Data analysis and synthesis

We will use RevMan5.3 software for meta-analysis. For dichotomous data (eg, effective and ineffective), we will calculate risk ratio and 95% confidence intervals (CIs). For continuous data, when the measurement method and unit are consistent, we will calculate the weighted mean difference and 95% CIs. When the measurement methods and units are inconsistent or the mean values of different experiments differ greatly, we will use the standardized mean difference and 95% CIs as the composite statistics.

#### 3.5.2 Investigation of heterogeneity

Heterogeneity was evaluated with *χ*2 test results and *I*^2^ statistics.^[19]^ If *P* ≤ .10 or *I*^2^ ≥ 50%, heterogeneity will be considered significant. At this point, we will use the random-effects model and conduct meta-regression or sensitivity analysis to judge the robustness of the combined results and find out the source of heterogeneity.

#### 3.5.3 Subgroup analysis

If there is significant heterogeneity in the included trials, we will identify the source of heterogeneity through subgroup analysis and manage the heterogeneity:

1. The duration and severity of VC.
2. Intervention features: unilateral varicose vein surgery or bilateral varicose vein surgery.
3. High duration of SDF.
4. Demographic characteristics of the patients: age, marital and family status, region, race, and ethnicity.
5. Follow-up time.

#### 3.5.4 Sensitivity analysis

A sensitivity analysis will be performed to test the robustness of the review result and to detect the source of heterogeneity. This can be done by excluding trials with a high risk of bias or eliminating each study individually. And, the impact of methodological quality, sample size, and missing data will be assessed. Then the analysis will be repeated after the exclusion of low methodological quality studies and the results compared with the previous meta-analysis.

#### 3.5.5 Grading the quality of evidence

Grading of Recommendations Assessment, Development, and Evaluation (GRADE) method^[20]^ will be performed to evaluate the level of confidence in regards to outcomes. It is based on 5 key domains: risk of bias, consistency, directness, precision, and publication bias. Two independent reviewers will assess these studies. In most cases, disagreements were resolved by discussion between the 2 reviewers. If disagreement remained after discussion, the third reviewer will be consulted before taking the final decision on the disagreements.

#### 3.5.6 Publication bias

Published bias will be measured by the funnel plot. If the result is indistinct, the Begg test and Egger test will be used (by STATA software 11.0).

#### 3.5.7 Reporting of the review

The methodological quality of the systematic review and meta-analysis will be standardized by each item of the AMSTAR-2 tool.^[21]^ And the results will be reported following the PRISMA statement.^[22]^

## 4 Discussion

After studying the relationship between VC and semen routine, more attention has been paid to the relationship between VC and sperm DNA. Now more and more studies have shown that VC is closely related to elevated SDF. Recently, VC repair has been used to treat male infertility and high SDF caused by spontaneous VC.^[16]^

Therefore, the purpose of this study was to evaluate the efficacy and safety of VC repair in the treatment of high SDF and male infertility. Through this study, more detailed observation and analysis of patients with VC surgery for male infertility can guide urologists to choose the mode of operation more reasonably and concretely and adopt the most appropriate treatment. There are some restrictions on this comment. As we are not good at other languages, the literature we search for is limited to Chinese and English, which will cause some prejudice. In addition, the limitation of the sample size also leads to the instability of the reliability of the conclusion.

## Author contributions

**Conceptualization:** Hangyu Yao

**Data curation:** Hangyu Yao, Fuhao Li

**Formal analysis:** Fuhao Li, Xianliang Qiu

**Methodology:** Hangyu Yao

**Project administration:** Hangyu Yao, Fuhao Li, Degui Chang

**Software:** Yuanjie Xu, Pengfei Xue

**Supervision:** Degui Chang

**Validation:** Hangyu Yao, Fuhao Li

**Writing – original draft:** Hangyu Yao

**Writing – review & editing:** Degui Chang

## Abbreviations

Abbreviations: CIs = confidence intervals, DFI = DNA fragmentation index, PRISMA = Preferred Reporting Items for Systematic Reviews and Meta-Analysis, SDF = sperm DNA fragmentation, VC = varicocele.
